# Identifying the minimum number of microsatellite loci needed to assess population genetic structure: A case study in fly culturing

**DOI:** 10.1080/19336934.2017.1396400

**Published:** 2017-12-01

**Authors:** Wolfgang Arthofer, Carina Heussler, Patrick Krapf, Birgit C. Schlick-Steiner, Florian M. Steiner

**Affiliations:** Molecular Ecology Group, Institute of Ecology, University of Innsbruck, Technikerstrasse 25, Innsbruck, Austria

**Keywords:** population genetics, microsatellite markers, population structure, genetic monitoring, loss of genetic variation, genetic drift, *Drosophila nigrosparsa*

## Abstract

Small, isolated populations are constantly threatened by loss of genetic diversity due to drift. Such situations are found, for instance, in laboratory culturing. In guarding against diversity loss, monitoring of potential changes in population structure is paramount; this monitoring is most often achieved using microsatellite markers, which can be costly in terms of time and money when many loci are scored in large numbers of individuals. Here, we present a case study reducing the number of microsatellites to the minimum necessary to correctly detect the population structure of two *Drosophila nigrosparsa* populations. The number of loci was gradually reduced from 11 to 1, using the Allelic Richness (AR) and Private Allelic Richness (PAR) as criteria for locus removal. The effect of each reduction step was evaluated by the number of genetic clusters detectable from the data and by the allocation of individuals to the clusters; in the latter, excluding ambiguous individuals was tested to reduce the rate of incorrect assignments. We demonstrate that more than 95% of the individuals can still be correctly assigned when using eight loci and that the major population structure is still visible when using two highly polymorphic loci. The differences between sorting the loci by AR and PAR were negligible. The method presented here will most efficiently reduce genotyping costs when small sets of loci (“core sets”) for long-time use in large-scale population screenings are compiled.

## Introduction

Culturing organisms in the laboratory is a frequently used method in many biological fields such as ecology, conservation biology, and evolutionary biology.^1,2^ With careful management, captive populations can maintain high genetic diversity over many generations. However, when population size is small, genetic variation may be lost due to genetic drift.^3,4^ A depletion of genetic variability can increase homozygosity within the population, which in turn may cause lower viability and fecundity, an effect termed inbreeding depression.^5^ Rapid identification of a loss of genetic variation within and/or among populations and generations is crucial for successful laboratory culturing.

One method to rapidly identify changes in genetic diversity is monitoring using molecular markers like microsatellites,^6^ single nucleotide polymorphisms,^7^ or whole-genome fingerprints.^8,9^ Such markers provide insight into genetic variation and evolutionary processes and allow identification of specimens and their populations of origin.^10,11^ A limiting factor for the use of genetic monitoring is that these techniques are complex, expensive, and time consuming.^12^ Here, we test a method to simplify genetic monitoring by a stepwise reduction of the number of molecular markers to the minimum needed to still detect the relevant signature of population structure.

In testing this method, we used microsatellite markers. Microsatellites were discovered in the early 1980s and are short and highly variable nucleotide tandem repeats in DNA sequences.^13–15^ The formerly tedious isolation of microsatellites for new species^16^ has, with the advent of next generation sequencing, become routine.^17,18^ Once isolated, these codominant markers are easily amplified by Polymerase Chain Reaction (PCR) and analyzed using capillary electrophoresis or, more recently, Illumina sequencing.^19^ Their mutation rate of 10^−6^ to 10^−2^ events per locus and generation^15,20^ qualifies them for the detection of drift effects within very few generations. While in the last years single nucleotide polymorphisms (SNPs) have gained in popularity relative to microsatellites due to their greater abundance in genomes and lower genotyping error rates, for analysis of, for instance, population size dynamics and population structure, microsatellites are on a per-locus basis two to 20 times more informative than SNPs.^21^ For many study designs, microsatellites provide an appropriate information density at substantially lower costs than genome-based approaches. Thus, microsatellites are considered to remain important genetic markers for years to come.^22^ While next generation sequencing based isolation of microsatellites now facilitates the use of arbitrarily large numbers of loci and thus very precise detection of molecular variation, in a scientific world with often limited resources the other way, that is, a cost efficient use of genetic markers, will often remain desirable. Fundamental factors for the expenses of a microsatellite-based study are the number of loci used and the resulting trade-off between accuracy and costs. In a recent study,^23^ we estimated the costs per locus and individual at ca. 3 Euro, based on multiplexing of three loci into one capillary electrophoresis run. Reducing the number of loci would thus diminish a study's costs in terms of money and time.

In detail, the purpose of this study was to evaluate a method determining how few loci are needed to still detect the short-term changes in genetic structure in a population threatened by loss of diversity. We used individuals of two populations of the cool-climate mountain fly *Drosophila nigrosparsa* Strobl, 1898.^24^ Native to the Alps and threatened by climate change, the fly has been in the focus of intense research^25–28^ including selection experiments for which it had to be kept in the laboratory. Permanent laboratory stocks were established in 2012 and genotyped with the full set of 11 variable microsatellite loci available for this species^29^ in Generation 0 and Generation 5. Then, the performance of various subsets of the full microsatellite set was evaluated. The criteria in evaluating the performance of a set of loci were (a) the number of genetic clusters retrieved and (b) the assignment of individuals to clusters. In applying criterion (a), we considered as optimum number of loci their lowest number at which still the correct number of clusters (i. e., the number of clusters retrieved with the full set of 11 loci) was identified. In applying criterion (b), the particular aim of a research has to be set; in the extremes, this can be either a maximum accuracy in assigning individuals to clusters or a maximum number of individuals that are assigned. We explored the trade-off that applies to the two aims, in that achieving the first aim comes at the cost of excluding individuals (here termed exclusion rate) and the second at the cost of losing accuracy in assigning individuals to clusters (incorrect assignment rate).^30^ In sorting loci for being reduced, we assessed both the Allelic Richness (AR) and the Private Allelic Richness (PAR) made accessible by each locus. AR is a proxy for the number of alleles per locus, a measure of genetic diversity that indicates the potential for adaptability and persistence of a population.^31^ PAR is a proxy for the number of alleles unique to a population and is a simple measure of genetic distinctiveness.^32,33^ In detail, we first removed the locus with the lowest values of AR or PAR and retained to the end the locus with the highest values.

## Materials and methods

Individuals of two natural populations of *Drosophila nigrosparsa* Strobl, 1898 were sampled at Kaserstattalm (Austria, 11.29°E 47.13°N, 2000 m above sea level, a.s.l.) and at Pfitscherjoch (Italy, 11.68°E 46.98°N, 2000 m a.s.l.) using fermented banana baits from July to August 2012.^34^ From each population, 100 males and 100 females were used to create a laboratory population, Kaserstattalm (henceforth K) and Pfitscherjoch (henceforth P). After oviposition, 31 field-caught female flies each of populations K and P were fixed in 96% ethanol and stored at −20°C for molecular analysis (Generation 0, henceforth K0 and P0). The laboratory populations K and P were kept and cultivated in quarantine for four generations to adapt flies to laboratory conditions and to eliminate potential diseases.^35^ Eggs were cultivated on malt medium (10 g agar, 1000 ml deionised water, 15 g dried yeast, 100 g ground malt, 3 ml methyl-4-hydroxybenzoate, 3.6 ml propionic acid, and 50 g ground maize),^36^ and adults were kept in inverted, transparent 0.3-l plastic cups with ventilation holes on petri-dishes with grape-juice agar (30 g agar, 1000 ml deionized water, 334 ml grape juice, 3.4 ml methyl-4-hydroxybenzoate, and 34 g sucrose).^37^ Flies were reared in environmental test chambers (MLR-352H-PE, Panasonic Healthcare Co., Japan) mimicking the diurnal temperature variation at 2000 m a.s.l. as well as the temperature at which the fly was found in the field at 2000 m a.s.l. in Tyrol in summer^38^ with a light:dark period of 16:8 hours and a humidity of 70% (Table S1). The fifth generation of laboratory populations K and P was randomly separated into eight lines. After oviposition, 31 females of each of the newly established lines were fixed in 96% ethanol and stored at −20°C for molecular analysis (Generation 5, henceforth K5 and P5).

DNA of individual flies was extracted using the Sigma GenElute Mammalian Genomic DNA Miniprep Kit (Sigma-Aldrich, St. Louis, USA) according to the manufacturer's instructions. Eleven species-specific microsatellite loci (DN16, DN31, DN35, DN36, DN37, DN39, DN40, DN41, DN45, DN48, DN49) were amplified via M13-tailed labelling.^29,39^ Amplifications were carried out in 5 µl reaction volume containing 1 × reaction buffer (Bioline, London, UK), 0.2 µM M13 primer, 0.02 µM M13-tailed forward primer, 0.2 µM reverse primer, 0.125 U MyTaq DNA polymerase (Bioline, London, UK), and 0.5 µl DNA extract on a UnoCycler 1200 (VWR, Radnor, USA). PCR conditions were 94°C for 2 min, followed by 35 cycles of 94°C for 30 s, T_a_ for 45 s, and 72°C for 1 min, followed by 72°C for 10 min. Locus-specific T_a_ was 48°C (DN16, DN36, DN40) and 55°C (DN31, DN35, DN37, DN39, DN41, DN45, DN49, DN55). Amplification success was checked by agarose gel electrophoresis. Capillary electrophoresis was performed by a commercial provider (CRC Sequencing Facility, Chicago, USA) using an ABI 3130 instrument (Applied Biosystems, Foster City, USA). The resulting traces were visualized using PeakScanner software v1.0 (Applied Biosystems, Foster City, USA) and scored manually. In total, microsatellite profiles of 558 individuals were generated (K0 = 31, K5 = 248, P0 = 31, P5 = 248).

Deviations from Hardy-Weinberg-Equilibrium (HWE), pairwise F_ST_, and Analyses of Molecular Variance (AMOVA) were computed in GenAlEx v6.41^40^. In the AMOVA, the generations (G0, G5) and the populations (K, P) were used as hierarchy levels in multiple combinations. Linkage disequilibrium (LD) was calculated using Arlequin v3.5 with 10,000 iterations.^41^ For HWE and LD, Bonferroni-Holm corrections for multiple testing were performed.^42^

To detect the genetic variation of K and P and to asses a potential effect of husbandry (loss of genetic variation from Generation 0 to Generation 5), AR and PAR were calculated for each population and generation using HP-Rare v1.0 assuming 52 genes,^32,43^ and significance (α = 0.05) was assessed by f- and two-sided t-tests in Excel 2013 (Microsoft Corp., Redmond, USA). HP-Rare uses rarefaction of alleles to compensate for differences in sample size, as larger samples are expected to display higher allele numbers.

STRUCTURE v2.3.3^44^ was used to identify the population structure of the complete data set. The admixture model with correlated allele frequencies was chosen as recommended for faint population structures.^45^ The number of clusters (K) assumed was set to [1, 8], and each value of K was run 10 times. The Markov Chain was run for 20,000 generations burnin and 180,000 generations data collection. The optimum K was calculated using the method described by Evanno, Regnaut & Goudet (2005).^46^

For further assessment of the minimum number of loci needed to distinguish between K and P flies, the individuals of Generation 5 were used. STRUCTURE analysis and search for best K were performed as described above with 496 individuals (K5 = 248, P5 = 248). The loci were then sorted by the mean AR value of the two populations; the locus with the lowest AR was removed and STRUCTURE analysis/best K search re-performed. Locus removal and data analysis were repeated until only the locus with the highest AR remained. The same procedure was applied on the dataset using PAR, resulting in each 10 reduced datasets for AR and PAR.

STRUCTURE analysis with 11 loci resulted in a best K of 2 (see Results and Discussion). From the 10 repetitions of this K, the individual cluster assignment of the run with the highest LnP(D) was used as benchmark for comparison with the STRUCTURE assignments of the reduced datasets. In detail, at K = 2, STRUCTURE provides for each individual a probability *p_A_* to belong to cluster A and a probability *p_B_* = 1 – *p_A_* to belong to cluster B. We considered an individual assigned to cluster A when its *p_A_* was larger than 0.5 plus a variable exclusion threshold value *x*. Accordingly, an individual was assigned to cluster B when *p_B_* > 0.5 + *x*. Individuals with *p_A_* or *p_B_* larger than 0.5 but smaller than or equal to 0.5 + *x* were considered as unassignable (U). Thresholds from *x* = 0.00 to *x* = 0.50 were used in 0.05-steps. As this algorithm for cluster assignment handles just K = 2, which is also the true number of clusters as discernible from the analysis using all data, also in the two reduced datasets where another value than two was suggested as best K (see Results), the STRUCTURE results for K = 2 were used for computation. On the individual level, this algorithm allowed three outcomes: (i) Correct assignment: An individual was assigned to the same cluster it had been assigned to when using all 11 loci. (ii) Incorrect assignment: An individual was assigned to the cluster opposite to the one it had been assigned to when using 11 loci. (iii) Exclusion: Due to an admixture value close to 0.5, no assignment was achieved. At *x* = 0.00, no exclusion occurred, while at *x* = 0.50 all individuals were excluded. Higher levels of *x* can be expected to reduce cases of incorrect assignment but also reduce the number of individuals assigned at all.

The assignment of each individual to A, B, and U in all loci-reduced datasets was compared with its assignment when using all data, and incorrect assignment rate (rate of individuals assigned to different clusters in benchmark and reduced dataset) and exclusion rate (rate of individuals unassignable in loci-reduced datasets) were calculated in Excel 2013.

## Results and discussion

**Figure 1.**
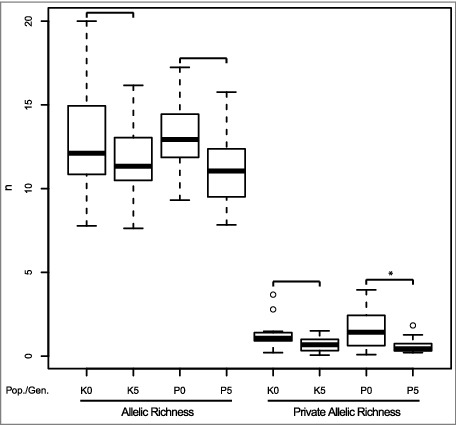
Allelic and Private Allelic Richness of populations Kaserstattalm (K) and Pfitscherjoch (P) in Generation 0 and Generation 5. Pairwise comparisons by two-sided t-tests are indicated by squared brackets. Significant differences (α = 0.05) are indicated by an asterisk.

Scoreable alleles were found in 89.6% (DN48) to 98.2% (DN40) of the individual amplicons (Table S2). The number of alleles per locus ranged from 12 to 25, with a mean of 19.36. AR per locus ranged from 7.63 (DN40 in K5) to 20.00 (DN39 in K0), PAR from 0.05 (DN40 in K5) to 3.95 (DN45 in P0). Over all loci, average AR was 13.38 for K0, 13.30 for P0, 11.77 for K5, and 11.18 for P5; average PAR was 0.99 for K0, 1.28 for P0, 0.45 for K5, and 0.51 for P5 (Fig. 1, Table S3). The loss of allelic diversity from Generation 0 to Generation 5 was significant for population P concerning PAR. This loss of diversity can likely be explained by drift acting on a relatively small laboratory population.^47^ Thus, the fly lines here are a suitable example for genetic pauperization, a situation that should be avoided in, for instance, laboratory culturing.

Significant deviations from HWE after Bonferroni-Holm correction were found in four and one loci in K0 and P0, respectively, and in seven and eight loci in K5 and P5, respectively (Table S4). Thus, the number of loci significantly deviating from HWE had increased from K0 to K5 and from P0 to P5. A similar trend emerged in LD, where the number of significantly linked locus pairs increased from K0 to K5 (3 to 30) and from P0 to P5 (0 to 21) (Table S5). Significant deviations from HWE and significant LD in wild populations like K0 and P0, which were collected in the field to establish the laboratory stocks, would indicate that non-neutral evolutionary forces like inbreeding, non-random mating, and/or selection are acting on the populations.^48^ However, both K0 and P0 samples were comparatively small, and more data are needed for reliable population genetic estimates of these wild populations of *D. nigrosparsa.* More importantly here, already after five generations in the laboratory, clear changes in HWE, LD, and diversity metrics were visible.

The AMOVA revealed significant but very low F_ST_ values ranging from 0.01 to 0.03 among the various combinations of populations and generations except between the two Generation 0 populations, for which the values were not significant (Table 1). The F_IS_ value ranged from 0.11 to 0.19 and was always significant in the various combinations. The percentage of variation between populations increased from 0.01% between K0 and P0 to 2.71% between K5 and P5. These results indicate a weak population differentiation, slightly increasing during five generations of laboratory culturing, and are in line with the loss of Allelic Richness observed between Generation 0 and 5.

**Table 1.** Results from Analyses of Molecular Variance and F-Statistics from various combinations of populations. df … degrees of freedom; reg … regions; pop … populations; ind … individuals; F_ST_ … fixation index of subpopulation compared with total population; F_IT_ … inbreeding coefficient of individuals relative to total population; F_IS_ … inbreeding coefficient of individuals relative to subpopulation; K0, K5 … populations from Kaserstattalm at Generation 0 and Generation 5; P0, P5 … populations from Pfitscherjoch at Generation 0 and Generation 5. All … all hierarchy levels (populations and generations).

AMOVA				Fixation indices		
Sample	Source of variation	df	% Variation	Index	Value	*p*
All	Among reg	1	1.37	F_ST_	0.02	0.01
	Among pop	2	1.07	F_IS_	0.15	0.01
	Among ind	554	14.82	F_IT_	0.17	0.01
	Within ind	558	82.74			
	Total	1115	100.00			
K0 & K5	Among pop	1	0.87	F_ST_	0.01	0.01
	Among ind	277	18.60	F_IS_	0.19	0.01
	Within ind	279	80.53	F_IT_	0.19	0.01
	Total	557	100.00			
P0 & P5	Among pop	1	1.27	F_ST_	0.01	0.01
	Among ind	277	11.38	F_IS_	0.12	0.01
	Within ind	279	87.35	F_IT_	0.13	0.01
	Total	557	100.00			
K0 & P0	Among pop	1	0.01	F_ST_	0.00	0.48
	Among ind	60	11.19	F_IS_	0.11	0.01
	Within ind	62	88.80	F_IT_	0.11	0.01
	Total	123	100.00			
K5 & P5	Among pop	1	2.71	F_ST_	0.03	0.01
	Among ind	494	14.56	F_IS_	0.15	0.01
	Within ind	496	82.73	F_IT_	0.17	0.01
	Total	991	100.00			

**Figure 2.**
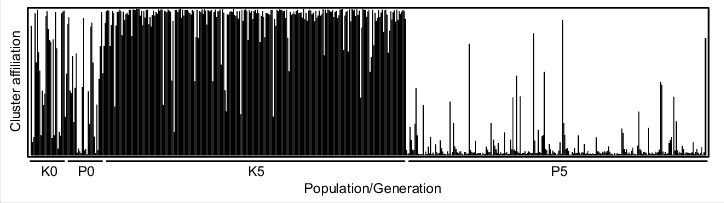
STRUCTURE results of the populations Kaserstattalm (K) and Pfitscherjoch (P) in Generation 0 and Generation 5. K = 2 was the most probable number of clusters. Loss of different alleles due to genetic drift during five generations laboratory rearing lead to increasing population differentiation.

STRUCTURE analysis of K0, P0, K5, and P5 resulted in a best K = 2. In Generation 0, single individuals assigned mainly to one of the two clusters, or intermediate, existed in both populations K and P. After five generations, some sorting had occurred, with one cluster dominated by individuals from K and the other by individuals from P (Fig. 2). This sorting was likely driven by genetic drift, which leads to loss of some (as seen in the analysis of AR) but not the same alleles in the two populations, as seen in the STRUCTURE plot. This sorting is far from being complete; still there are several ‘K-type’ individuals in P5 and vice versa, and many intermediates. By systematically reducing the number of microsatellite loci, we then tested whether an individual was still assigned to the same cluster as with the full set of 11 loci.

**Figure 3.**
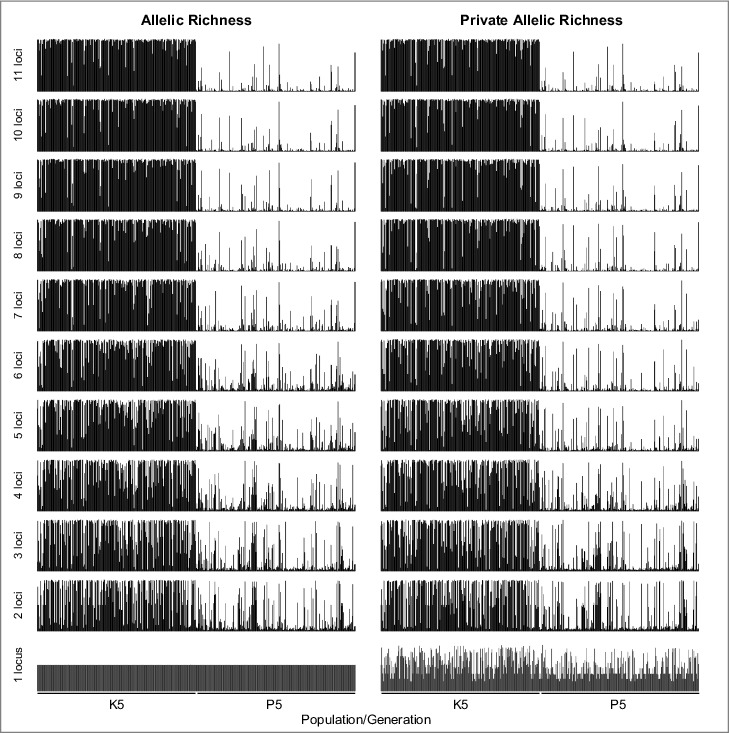
STRUCTURE results after sequential removal of loci, sorted according to Allelic (AR) and Private Allelic Richness (PAR). Plots in which the estimate for the best K deviated from 2 are printed in grey. While the separation quality gradually deteriorated, the major population structure was still visible when only the two loci with highest AR or PAR are used.

**Figure 4.**
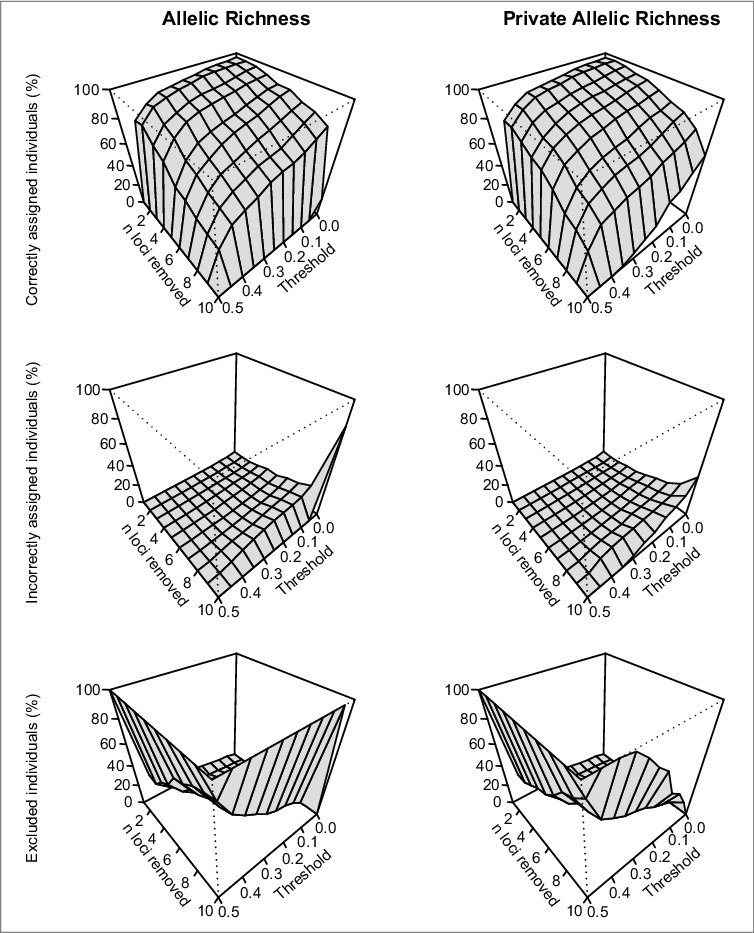
Based on the STRUCTURE results, individuals genotyped at one to ten loci were assigned to two clusters. Individuals with intermediate probabilities to belong to one cluster were excluded using a variable threshold *x*; *x* = 0 results in no exclusions, *x* = 0.5 excludes all individuals. The cluster assignment with 11 loci was used as a benchmark. Each individual could be either correctly assigned (i. e., in the same cluster as with 11 loci; upper row of plots), incorrectly assigned (i.e., in the other cluster than with 11 loci; middle row), or excluded from assignment based on the threshold value (lower row). High thresholds minimize incorrect assignments at the cost of many excluded individuals.

The sequence of locus removal is given in Table 2, and the resulting STRUCTURE plots for K = 2 are given in Fig. 3. The method of Evanno et al.^46^ suggested K = 2 for all reduced datasets except for those where just one locus remained. The detailed results of assignment accuracy by stepwise exclusion of microsatellite loci are given in Fig. 4 and Supplemental Tables S6-S9. As expected, the number of correctly assigned individuals decreased both with fewer loci and higher thresholds. A trade-off between accepting incorrect assignments versus accepting high numbers of excluded individuals is inherent in this approach. Considering the often-used error level of 5%, a correct assignment was still possible with eight loci both when sorted by AR and PAR. With fewer than eight loci, also the rate of incorrect assignments exceeded 5% when low thresholds were applied. Remarkably, a correct assignment of more than 80% of the individuals was still possible with as few as three loci. Correct assignment dropped to 76.6% and 74.6% for AR and PAR, respectively, when data of only two loci were used, although these were the most polymorphic markers. Any population structure vanished when just the single locus with the highest AR and PAR was used (Fig. 3). The differences between sorting the loci by AR and PAR were negligible.

**Table 2.** Sequence of locus removal for microsatellite data on population Kaserstattalm in Generation 5. Loci were sorted from lowest to highest Allelic Richness (AR) and Private Allelic Richness (PAR) and sequentially removed. The number of alleles remaining and the value suggested for the best K using the method of Evanno et al. (2005) are given. In AR, locus DN16 (AR = 15.96) remained as last marker, in PAR, locus DN41 (PAR = 1.38).

Removal by AR value					Removal by PAR value				
n loci	removed locus	AR of removed locus	n alleles in dataset	best K	n loci	removed locus	PAR of removed locus	n alleles in dataset	best K
11	none	—	193	2	11	none	—	193	2
10	DN40	7.73	174	2	10	DN40	0.19	174	2
9	DN45	9.13	159	2	9	DN31	0.27	158	2
8	DN35	9.83	135	2	8	DN45	0.32	143	2
7	DN49	10.24	121	2	7	DN48	0.33	122	2
6	DN48	10.64	100	2	6	DN36	0.59	110	2
5	DN31	11.19	84	2	5	DN39	0.59	94	2
4	DN41	12.12	69	2	4	DN35	0.74	70	2
3	DN39	12.90	53	2	3	DN16	0.75	47	2
2	DN36	12.94	41	2	2	DN49	0.92	33	2
1	DN37	13.54	23	7	1	DN37	1.25	15	4

Screening for loss of genetic diversity is of high importance for laboratory culturing and will often require the screening of many individuals.^49^ While the costs of genotyping a single microsatellite locus are around 3 Euro,^23^ costs pile up with adding loci, especially when the population to be screened is large. In this study, we have provided the proof of concept for a method of reducing the number of loci while still detecting genetic structure and its changes across generations. This method is especially promising when core sets, that is, smaller sets of markers selected from a large set of microsatellite markers, are to be established for broad-scale screening.^50–52^ The particular number of loci one removes will depend on whether maximum accuracy or information on a maximum number of individuals is desired. Using AR and PAR as a criterion for the removal of loci yields similar results. In adopting this method, long-time and large-scale population genetic investigations will benefit from a substantial reduction of costs in terms of money and processing time.

## Supplementary Material

suppl_Mat.zip

## Notes

**List of abbreviations**
α: Alpha
*AMOVA*: Analysis of molecular variance
*°C*: Degree Celsius
µ*l*: Microlitre
µ*M*: Micromol
*a.s.l.*: Above sea level
*AR*: Allelic Richness
*df*: Degrees of freedom
*DNA*: Deoxyribonucleic acid
*E*: East
*et al.*: et alii
*F_IS_*: Inbreeding coefficient of individuals relative to subpopulation
*F_IT_*: Inbreeding coefficient of individuals relative to total population
*F_ST_*: Fixation index of subpopulation compared with total population
*g*: Gram (mass unit)
*HWE*: Hardy-Weinberg equilibrium
*i. e.*: id est
*K*: Kaserstattalm (population)
*K*: Number of clusters (STRUCTURE software)
*K0*: Individuals from Kaserstattalm in Generation 0
*K5*: Individuals from Kaserstattalm in Generation 5
*LD*: Linkage disequilibrium
*LnP(D)*: Mean posterior probability (STRUCTURE software)
*ml*: Millilitre
*n*: Number
*N*: North
*p*: Probability
*P*: Pfitscherjoch (population)
*P0*: Individuals from Pfitscherjoch in Generation 0
*P5*: Individuals from Pfitscherjoch in Generation 5
*PAR*: Private Allelic Richness
*PCR*: Polymerase chain reaction
*s*: Second
*SNP*: Single nucleotide polymorphism
*T_a_*: Annealing Temperature
*U*: Unit

## References

[1] Wang J, Ryman N Genetic effects of multiple generations of supportive breeding. Conserv Biol. 2001;15:1619–31. doi:10.1046/j.1523-1739.2001.00173.x.

[2] Clutton-Brock T, Sheldon BC Individuals and populations: the role of long-term, individual-based studies of animals in ecology and evolutionary biology. Trends Ecol Evol. 2010;25:562–73. doi:10.1016/j.tree.2010.08.002. PMID:20828863. 20828863

[3] Lacy RC Loss of genetic diversity from managed populations: interacting effects of drift, mutation, immigration, selection, and population subdivision. Conserv Biol. 1987;1:143–58. doi:10.1111/j.1523-1739.1987.tb00023.x.

[4] Kristensen TN, Sørensen AC Inbreeding – lessons from animal breeding, evolutionary biology and conservation genetics. Anim Sci. 2005;80:121–33. doi:10.1079/ASC41960121.

[5] Charlesworth D, Charlesworth B Inbreeding depression and its evolutionary consequences. Annu Rev Ecol Syst. 1987;18:237–68. doi:10.1146/annurev.es.18.110187.001321.

[6] Jarne P, Lagoda P Microsatellites, from molecules to populations and back. Trends Ecol Evol. 1996;11:424–9. doi:10.1016/0169-5347(96)10049-5. PMID:21237902. 21237902

[7] Vignal A, Milan D, SanCristobal M, Eggen A A review on SNP and other types of molecular markers and their use in animal genetics. Genet Sel Evol. 2002;34:275–305. doi:10.1186/1297-9686-34-3-275. PMID:12081799. 12081799 PMC2705447

[8] Vos P, Hogers R, Bleeker M, Reijans M, van de Lee T, Hornes M, Frijters A, Pot J, Peleman J, Kuiper M AFLP: a new technique for DNA fingerprinting. Nucleic Acids Res. 1995;23:4407–14. doi:10.1093/nar/23.21.4407. PMID:7501463. 7501463 PMC307397

[9] Davey JL, Blaxter MW RADSeq: next-generation population genetics. Brief Funct Genomics. 2010;9:416–23. doi:10.1093/bfgp/elq031. PMID:21266344. 21266344 PMC3080771

[10] Schwartz MK, Luikart G, Waples RS Genetic monitoring as a promising tool for conservation and management. Trends Ecol Evol. 2007;22:25–33. doi:10.1016/j.tree.2006.08.009. PMID:16962204. 16962204

[11] Palsbøll PJ, Zachariah Peery M, Olsen MT, Beissinger SR, Bérubé M Inferring recent historic abundance from current genetic diversity. Mol Ecol. 2013;22:22–40. doi:10.1111/mec.12094. PMID:23181682. 23181682

[12] Kurtz TW, Montano M, Chan L, Kabra P Molecular evidence of genetic heterogeneity in Wistar-Kyoto rats: implications for research with the spontaneously hypertensive rat. Hypertension. 1989;13:188–92. doi:10.1161/01.HYP.13.2.188. PMID:2914738. 2914738

[13] Balloux F, Lugon-Moulin N The estimation of population differentiation with microsatellite markers. Mol Ecol. 2002;11:155–65. doi:10.1046/j.0962-1083.2001.01436.x. PMID:11856418. 11856418

[14] Ellegren H. Microsatellites: simple sequences with complex evolution. Nat Rev Genet. 2004;5:435–45. doi:10.1038/nrg1348. PMID:15153996. 15153996

[15] Bhargava A, Fuentes FF Mutational dynamics of microsatellites. Mol Biotechnol. 2010;44:250–66. doi:10.1007/s12033-009-9230-4. PMID:20012711. 20012711

[16] Zane L, Bargelloni L, Patarnello T Strategies for microsatellite isolation: a review. Mol Ecol. 2002;11:1–16. doi:10.1046/j.0962-1083.2001.01418.x. PMID:11903900. 11903900

[17] Castoe TA, Poole AW, Gu W, JASON de KONING AP, Daza JM, Smith EN, Pollock DD Rapid identification of thousands of copperhead snake (*Agkistrodon contortrix*) microsatellite loci from modest amounts of 454 shotgun genome sequence. Mol Ecol Resour. 2010;10:341–7. doi:10.1111/j.1755-0998.2009.02750.x. PMID:21565030. 21565030 PMC5172459

[18] Castoe TA, Poole AW, de Koning APJ, Jones KL, Tomback DF, Oyler-McCance SJ, Fike JA, Lance SL, Streicher JW, Smith EN, et al. Rapid microsatellite identification from Illumina paired-end genomic sequencing in two birds and a snake. PLOS ONE. 2012;7:e30953. doi:10.1371/journal.pone.0030953. PMID:22348032. 22348032 PMC3279355

[19] Zhan L, Paterson IG, Fraser BA, Watson B, Bradbury IR, Nadukkalam Ravindran P, Reznick D, Beiko RG, Bentzen P megasat: automated inference of microsatellite genotypes from sequence data. Mol Ecol Resour. 2017;17:247–56. doi:10.1111/1755-0998.12561. PMID:27333119. 27333119

[20] Schlick-Steiner BC, Arthofer W, Moder K, Steiner FM Recent insertion/deletion (reINDEL) mutations: increasing awareness to boost molecular-based research in ecology and evolution. Ecol Evol. 2015;5:24–35. doi:10.1002/ece3.1330. PMID:25628861. 25628861 PMC4298431

[21] Hess JE, Matala AP, Narum SR Comparison of SNPs and microsatellites for fine-scale application of genetic stock identification of Chinook salmon in the Columbia River Basin. Mol Ecol Resour. 2011;11:137–49. doi:10.1111/j.1755-0998.2010.02958.x. PMID:21429170. 21429170

[22] Hodel RGJ, Segovia-Salcedo MC, Landis JB, Crowl AA, Sun M, Liu X, Gitzendanner MA, Douglas NA, Germain-Aubrey CC, Chen S, et al. The report of my death was an exaggeration: a review for researchers using microsatellites in the 21st century. Appl Plant Sci. 2016;4:1600025. doi:10.3732/apps.1600025. PMC4915923 27347456

[23] Arthofer W, Steiner FM, Schlick-Steiner BC Rapid and cost-effective screening of newly identified microsatellite loci by high-resolution melting analysis. Mol Genet Genomics. 2011;286:225–35. doi:10.1007/s00438-011-0641-0. PMID:21847526. 21847526

[24] Bächli G, Burla H Insecta Helvetica 7: Diptera, Drosophilidae. Zurich: Schweizer Entomologische Gesellschaft;1985.

[25] Consortium Genomic Resources Development, W Arthofer, Banbury BL, Carneiro M, Cicconardi F, Duda TF, Harris RB, Kang DS, Leaché AD, Nolte V, et al. Genomic Resources Notes Accepted 1 August 2014–30 September 2014. Mol Ecol Resour. 2015;15:228–9. doi:10.1111/1755-0998.12340. PMID:25424247. 25424247

[26] Cicconardi F, Di Marino D, Olimpieri PP, Arthofer W, Schlick-Steiner BC, Steiner FM Chemosensory adaptations of the mountain fly *Drosophila nigrosparsa* (Insecta: Diptera) through genomics’ and structural biology's lenses. Sci Rep. 2017;7:43770. doi:10.1038/srep43770. PMID:28256589. 28256589 PMC5335605

[27] Cicconardi F, Marcatili P, Arthofer W, Schlick-Steiner BC, Steiner FM Positive diversifying selection is a pervasive adaptive force throughout the *Drosophila* radiation. Mol Phylogenet Evol. 2017;112:230–43. doi:10.1016/j.ympev.2017.04.023. PMID:28458014. 28458014

[28] Kinzner M-C, Tratter M, Bächli G, Kirchmair M, Kaufmann R, Arthofer W, Schlick-Steiner BC, Steiner FM Oviposition substrate of the mountain fly *Drosophila nigrosparsa* (Diptera: Drosophilidae). PLOS ONE. 2016;11:e0165743. doi:10.1371/journal.pone.0165743. PMID:27788257. 27788257 PMC5082818

[29] Arthofer W, Heussler C, Krapf P, Schlick-Steiner BC, Steiner FM Sixteen new microsatellite loci for *Drosophila nigrosparsa*, an emerging system for studying temperature effects in mountain ecosystems. Mol Ecol Resour. 2013;13:966–8. PMID:23937578.23937578

[30] Kinzner M-C, Wagner HC, Peskoller A, Moder K, Dowell FE, Arthofer W, Schlick-Steiner BC, Steiner FM A near-infrared spectroscopy routine for unambiguous identification of cryptic ant species. PeerJ. 2015;3:e991. doi:10.7717/peerj.991. PMID:26734510. 26734510 PMC4699785

[31] Leberg PL Estimating allelic richness: effects of sample size and bottlenecks. Mol Ecol. 2002;11:2445–9. doi:10.1046/j.1365-294X.2002.01612.x. PMID:12406254. 12406254

[32] Kalinowski ST Counting alleles with rarefaction: private alleles and hierarchical sampling designs. Conserv Genet. 2004;5:539–43. doi:10.1023/B:COGE.0000041021.91777.1a.

[33] Moges AD, Admassu B, Belew D, Yesuf M, Njuguna J, Kyalo M, Ghimire SR Development of microsatellite markers and analysis of genetic diversity and population structure of *Colletotrichum gloeosporioides* from Ethiopia. PLOS ONE. 2016;11:e0151257. doi:10.1371/journal.pone.0151257. PMID:26978654. 26978654 PMC4792483

[34] Dobzhansky T, Cooper DM, Phaff HJ, Knapp EP, Carson HL Differential attraction of species of *Drosophila* to different species of Yeasts. Ecology 1956;37:544–50. doi:10.2307/1930178.

[35] Ashburner M, Roote J Maintenance of a *Drosophila* Laboratory: General Procedures. Cold Spring Harb Protoc. 2007;2007:pdb.ip35. doi:10.1101/pdb.ip35. 21357030

[36] Lakovaara S Malt as a culture medium for *Drosophila* species. Drosoph Inf Serv 1969;44:128.

[37] Sullivan W, Ashburner M, Hawley R *Drosophila* Protocols. Woodbury, NY: Cold Spring Harbor Laboratory Press;2008.

[38] Arthofer W, Decristoforo C, Schlick-Steiner BC, Steiner FM Ultra-low activities of a common radioisotope for permission-free tracking of a drosophilid fly in its natural habitat. Sci Rep. 2016;6:36506. doi:10.1038/srep36506. PMID:27812000. 27812000 PMC5095666

[39] Boutin-Ganache I, Rasposo M, Raymond M, Dschepper C M13-tailed primers improve the readability and usability of microsatellite analyses performed with two different allele-sizing methods. Biotechniques. 2001;31:25–8. 11464515

[40] Peakall R, Smouse PE GenAlEx 6.5: genetic analysis in Excel. Population genetic software for teaching and research—an update. Bioinformatics. 2012;28:2537–9. 22820204 10.1093/bioinformatics/bts460 PMC3463245

[41] Excoffier L, Lischer HEL Arlequin suite ver 3.5: a new series of programs to perform population genetics analyses under Linux and Windows. Mol Ecol Resour. 2010;10:564–7. doi:10.1111/j.1755-0998.2010.02847.x. PMID:21565059. 21565059

[42] Armstrong RA When to use the Bonferroni correction. Ophthalmic Physiol Opt. 2014;34:502–8. doi:10.1111/opo.12131. PMID:24697967. 24697967

[43] Kalinowski ST HP-Rare 1.0: a computer program for performing rarefaction on measures of allelic richness. Mol Ecol Notes. 2005;5:187–9. doi:10.1111/j.1471-8286.2004.00845.x.

[44] Pritchard JK, Stephens M, Donnelly P Inference of population structure using multilocus genotype data. Genetics. 2000;155:945–59. PMID:10835412. 10835412 10.1093/genetics/155.2.945 PMC1461096

[45] Falush D, Stephens M, Pritchard JK Inference of population structure using multilocus genotype data: linked loci and correlated allele frequencies. Genetics. 2003;164:1567–87. PMID:12930761. 12930761 10.1093/genetics/164.4.1567 PMC1462648

[46] Evanno G, Regnaut S, Goudet J Detecting the number of clusters of individuals using the software structure: a simulation study. Mol Ecol. 2005;14:2611–20. doi:10.1111/j.1365-294X.2005.02553.x. PMID:15969739. 15969739

[47] Greenbaum G, Templeton AR, Zarmi Y, Bar-David S Allelic richness following population founding events – a stochastic modeling framework incorporating gene flow and genetic drift. PLOS ONE. 2014;9:e115203. doi:10.1371/journal.pone.0115203. PMID:25526062. 25526062 PMC4272294

[48] Keller LF, Waller DM Inbreeding effects in wild populations. Trends Ecol Evol. 2002;17:230–41. doi:10.1016/S0169-5347(02)02489-8.

[49] Toro MA, Caballero A Characterization and conservation of genetic diversity in subdivided populations. Philos Trans R Soc Lond B Biol Sci. 2005;360:1367–78. doi:10.1098/rstb.2005.1680. PMID:16048780. 16048780 PMC1569508

[50] Kim KS, Stolz U, Miller NJ, Waits ER, Guillemaud T, Sumerford DV, Sappington TW A core set of microsatellite markers for Western Corn Rootworm (Coleoptera: Chrysomelidae) population genetics studies. Environ Entomol. 2008;37:293–300. doi:10.1093/ee/37.2.293. PMID:18419899. 18419899

[51] Bryda EC, Riley LK Multiplex microsatellite marker panels for genetic monitoring of common rat strains. J Am Assoc Lab Anim Sci JAALAS. 2008;47:37–41. PMID:18459711. 18459711 PMC2654014

[52] Politov DV, Belokon MM, Belokon YS, Polyakova TA, Shatokhina AV, Mudrik EA, Azarova AB, Filippov MV, Shestibratov KA Application of microsatellite loci for molecular identification of elite genotypes, analysis of clonality, and genetic diversity in Aspen *Populus tremula* L. (Salicaceae). Int J Plant Genomics. 2015;2015:1–11. doi:10.1155/2015/261518. PMC4707373 26823661

[53] Rice WR Analyzing tables of statistical tests. Evolution. 1989;43:223–5. doi:10.1111/j.1558-5646.1989.tb04220.x. PMID:28568501. 28568501

